# High-value biomass from microalgae production platforms: strategies and progress based on carbon metabolism and energy conversion

**DOI:** 10.1186/s13068-018-1225-6

**Published:** 2018-08-20

**Authors:** Han Sun, Weiyang Zhao, Xuemei Mao, Yuelian Li, Tao Wu, Feng Chen

**Affiliations:** 1 Institute for Food & Bioresource Engineering, College of Engineering, Peking University, Beijing, 100871 China; 2 BIC-ESAT, College of Engineering, Peking University, Beijing, 100871 China

**Keywords:** Microalgae, Carbon metabolism, Photosynthetic efficiency, Lipid, Carotenoid, Engineering strategy

## Abstract

Microalgae are capable of producing sustainable bioproducts and biofuels by using carbon dioxide or other carbon substances in various cultivation modes. It is of great significance to exploit microalgae for the economical viability of biofuels and the revenues from high-value bioproducts. However, the industrial performance of microalgae is still challenged with potential conflict between cost of microalgae cultivation and revenues from them, which is mainly ascribed to the lack of comprehensive understanding of carbon metabolism and energy conversion. In this review, we provide an overview of the recent advances in carbon and energy fluxes of light-dependent reaction, Calvin–Benson–Bassham cycle, tricarboxylic acid cycle, glycolysis pathway and processes of product biosynthesis in microalgae, with focus on the increased photosynthetic and carbon efficiencies. Recent strategies for the enhanced production of bioproducts and biofuels from microalgae are discussed in detail. Approaches to alter microbial physiology by controlling light, nutrient and other environmental conditions have the advantages of increasing biomass concentration and product yield through the efficient carbon conversion. Engineering strategies by regulating carbon partitioning and energy route are capable of improving the efficiencies of photosynthesis and carbon conversion, which consequently realize high-value biomass. The coordination of carbon and energy fluxes is emerging as the potential strategy to increase efficiency of carbon fixation and product biosynthesis. To achieve more desirable high-value products, coordination of multi-stage cultivation with engineering and stress-based strategies occupies significant positions in a long term.

## Background

The per capita energy demand is increasing with the development of society, while the consumption of fossil fuels available to a world is defined by the aggravating environment [1]. The advantage of biomass route lies in the fact that solar energy is trapped into organic material and potentially converted to biofuels (e.g., lipid, ethanol) over time and distance. Microalgae, therefore, are considered as a promising feedstock for biofuels and various high-value bioproducts for their high photosynthetic efficiency and short life cycle [2]. Compared with higher plants, microalgae have the greater ability to fix carbon dioxide (CO_2_) and then convert CO_2_ into biomass and potentially into products of interest. Furthermore, their less requirements for arable land and freshwater make them attractive for commercial exploitation. However, thus far, the biotechnological application in microalgae has been limited under industrial conditions for the lack of comprehensive understanding of carbon metabolism and energy conversion. Having considered the interest in industrial settings, many institutions and companies designed and proposed new strategies to improve the industrial performance of microalgae, such as utilizing light-emitting diode (LED) light, optimizing culture modes and modifying carbon fixation pathways and photosystems [3–5].

**Fig. 1:**
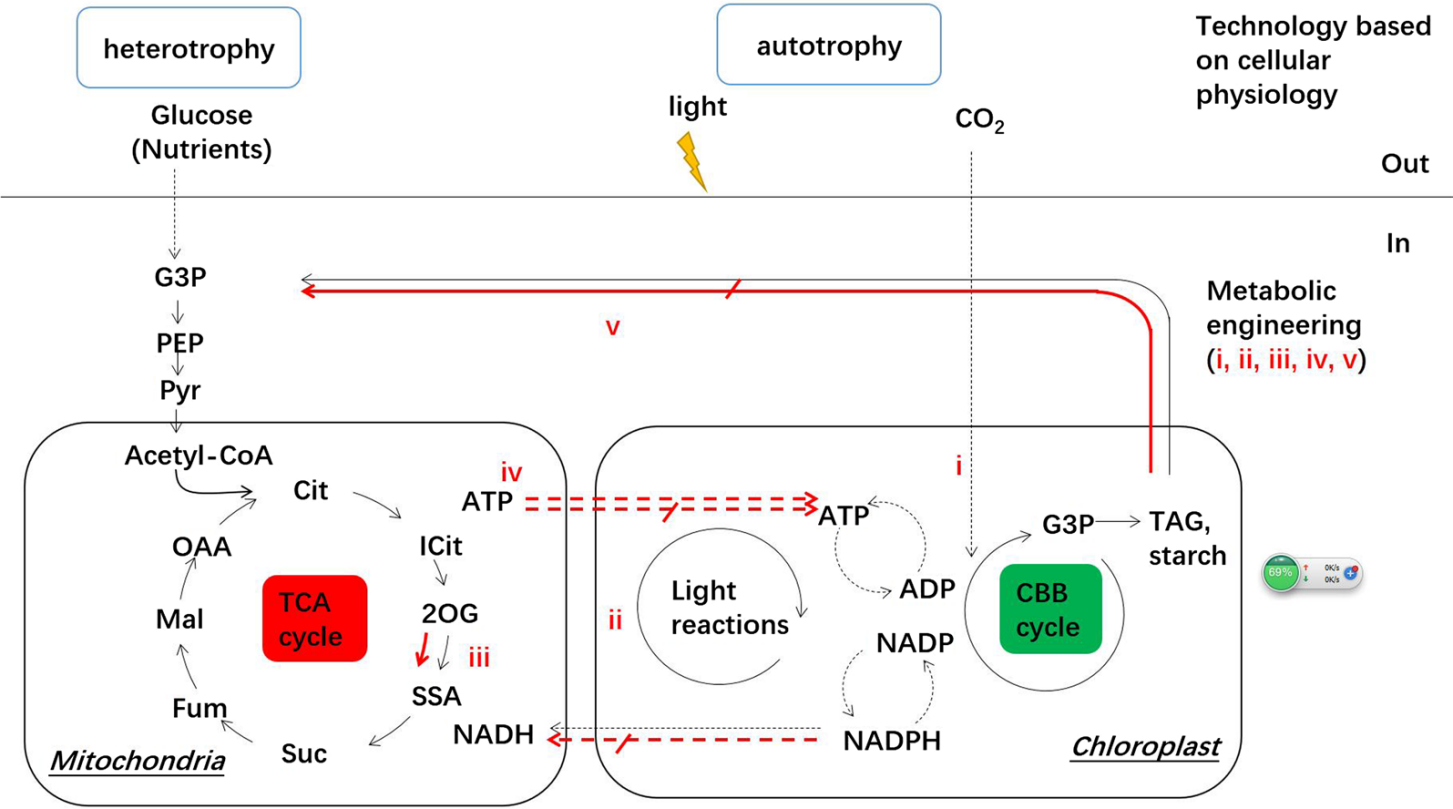
Carbon metabolism and energy conversion in glycolysis, CBB and TCA. In carbon fixation of microalgae, the metabolic engineering is traditionally focused on efficiency of CBB cycle and light reactions in chloroplast (i and ii). Advances have noticed that engineering TCA cycle increases the carbon fixation (iii and iv). Coupling carbon and energy fluxes have proposed as the trends of metabolic engineering in microalgae (v). The traditional technology based on cellular physiology focuses on conditions of CO_2_, light and nutrient in microalgae cultivation. The carbon metabolites: *G3P* glyceraldehyde-3-phosphate, *PYR* pyruvate, *PEP* phosphoenolpyruvate, *OAA* oxaloacetate, *MAL* malate, *FUM* fumarate, *SSA* Succinyl semialdehyde, *SUC* succinate, *2OG* 2-oxoglutarate, *CIT* citrate, *G3P* glyceraldehyde 3-phosphate

To improve the utilization of substrate and light, cultivation systems of microalgae, such as cultivation modes and bioreactors, have been modified based on the microbial physiology. Such systems have (partially) successfully provided sufficient, but not excess nutrients and photons to lengthen the logarithmic growth phase of microalgae. In addition, heterotrophic cultivation of microalgae has been introduced to increase the product yields (Fig. 1). Although the supply of energy or substance is sufficient in culturing environment, microalgae evolve their systems and metabolic pathways to produce biomass with less conversion efficiency, around 2–6% photosynthetic efficiency to fix CO_2_ and 5–10% allocated carbon to synthesize fuels and chemicals in natural conditions [6]. Therefore, as an alternative, genetic and metabolic engineering technologies of microalgae are considered to pave the way for efficient microalgae production platforms as more information of critical pathways are revealed, such as Calvin–Benson–Bassham (CBB) cycle, Embden-Meyerhof pathway (EMP), the pentose phosphate (PP) pathway and the tricarboxylic acid (TCA) cycle (Fig. 1) [7, 8]. To increase the photosynthetic efficiency, manipulations of the CBB cycle (i) and chloroplastic electron transport chain (ii) are of great interest by improving CO_2_ fixation and light-harvesting efficiencies, respectively. As mitochondria and chloroplast have exchanges of substance and energy, the regulation of TCA cycle may be feasible to enhance photosynthetic activity and redistribute the carbon flux (iii, iv and v) [7, 9]. These genetic and metabolic engineering technologies provide useful insights into possibilities and challenges to couple carbon flux and energy flux to further increase the photosynthetic efficiency.

Our focus here is on recent biotechnological applications in microalgae to increase biomass and product yields. First, we will describe the carbon metabolism in biomass accumulation and product formation and introduce utilization of solar energy flux in microalgae. We will focus primarily on carbon allocation and energy utilization of microalgae in natural conditions. Then, we will introduce the traditional technologies to increase biomass and product yields based on cellular physiology in microalgae. Third, we will discuss attractive strategies that have the great capacity to increase biomass and product yields by allocating carbon and rerouting energy, and assess the potential of new strategies of coupling carbon and energy flux. Finally, we will highlight metabolic engineering of microalgae that enables higher biomass concentration and product yield and discuss relatively underdeveloped strategies to consolidate contacts between carbon and energy fluxes.

## Carbon metabolism in microalgae

Microalgae evolve their systems and metabolic pathways to orchestrate growth characteristics and composition in different cultivation conditions. The biomass and product yields are well known to depend on the cultivation conditions and can be enhanced by regulating carbon metabolism, which is a theoretically carbon–neutral alternative in metabolic network. The theoretical biomass yield of microalgae was reported as 100–200 g dry weight m^−2^ day^−1^ and the practical productivity rate was 15–30 g dry weight m^−2^ day^−1^ [6], with the high content of protein or carbohydrate as the primary metabolites. After completing a certain amount of synthetic work to reserve the materials and energy, the cell acts to divide into the daughter cells by undergoing the metabolic pathways such as DNA replication and nuclear division. In microalgae, the metabolism of reserving materials and energy is prior to steady growth and division of microalgae, which means once the nutrient is limited in the environment, the cell stops to grow even though the substance and energy in cell are sufficient for the reproductive sequence. Only after providing nutrients in the nutrient-poor environment, the cell is activated to grow larger and subsequently divide into more or less. In addition, the cultivation environment is changing as the biomass enhances and nutrient concentration decreases [2], which enlarges energy losses from light harvesting and the subsequent processes of microalgae cultivation. Aside from the energy losses, more materials and energy are required to coordinate the environment. This results in an inhibition of high-density cultivation [2]. Therefore, understanding of carbon metabolism in biomass accumulation and product formation is crucial to explore new strategies to increase the industrial performance of microalgae.

### Photoautotrophic cultivation

Photosynthetic CO_2_ fixation is usually performed by higher plants and microalgae; yet reaction center (RC) in microalgae suffices to possess a greater ability to fix CO_2_. In CBB cycle, CO_2_ is captured by ribulose-1, 5-bisphosphate carboxylase oxygenase (Rubisco), which has a low specific affinity for CO_2_ and is prone to catalyze the oxygenation of the substrate [10]. In the oxygenase reaction, the product of 2-phosphoglycolate undergoes complex changes to enter into photorespiration, which releases CO_2_ and requires energy input. As a result, the efficiency of carbon fixation and solar energy conversion is suppressed. In microalgae, various carbon-concentration mechanisms (CCMs) are present to increase the local CO_2_ concentration around Rubisco [11].

The tolerance of microalgae against carbon concentration differs and maybe species-specific; the form of CO_2_ (H_2_CO_3_, HCO_3_^−^, CO_3_^2−^, CO_2_) in media also plays different roles to increase productivity. The content of 0.03% CO_2_ (V/V) in air is not sufficient for microalgae growth in a relative short time [12]. It was reported that import of flue gas containing 2–15% CO_2_ (V/V) into bioreactor and pond has different effects on specific microalgae species [12, 13]. Traditionally, CO_2_ levels as low as 2–5% are saturating to exert enhancing effect on growth and photosynthesis of microalgae, while higher CO_2_ levels often displayed deleterious effects. Higher CO_2_ levels are believed to disturb cellular pH homeostasis, thus to upregulate H^+^-ATPases, restrain CCM and adjust fatty acid composition of membranes. The CO_2_-tolerance level of used microalgae should be clear in mass cultivation as the optimal CO_2_ feeding rate is limited by the level.

### Heterotrophic cultivation

Although microalgae have greater potential to utilize solar energy and environmental CO_2_ than higher plants, the growth rate of microalgae is still limited by the efficiency of light harvesting, energy conversation and CO_2_ fixation. The introduction of sufficient natural and artificial light to allow massive growth is the main goal and a limiting factor for cultivation. To eliminate the requirement for light and offer the possibility of greatly increasing cell concentration, heterotrophic cultivation has been pointed out as promising efficient and sustainable approach for some microalgae to produce biofuels and bioproducts by using carbon substances as the sole carbon and energy source. The metabolism of respiration is applied to produce energy. The respiration rates, intimately geared to the growth and division, are determined by the oxidization of organic substrates of the given microalgae. The frequently used carbon sources consist of glucose, glycerol and acetate. Glucose is the most commonly used organic carbon source in microalgae cultivation as it produces more energy per mole compared to other substrates. During the oxidative assimilation of glucose, EMP and PP pathway are the only two pathways shown in algae from aerobic glycolysis. Glucose metabolized by microalgae is mainly via PP pathway in dark and via EMP in light conditions in cytosol [14]. Under some heterotrophic growth conditions, the growth rate, lipid content and ATP of microalgae are higher than those under photoautotrophic conditions and mainly depend on the species and strain used. The microalgae grow steadily and rapidly in nutrient-rich environment by high level of system control, reaching biomass of around 50–100 g L^−1^, which is higher than that achieved in photoautotrophic cultivation [14].

### Products from microalgae

Following the assimilation of CO_2_ or carbon substances, these carbon sources are potentially metabolized to biofuels and bioproducts, which are widely used in food, pharmaceutical and feeding industries as well as in energy area. Carotenoids and fatty acids from microalgae have got increasing attention as their potential health benefits are being revealed [15–17]. In addition, microalgae are pointed as the perfect source to produce biodiesel and bioethanol as they convert CO_2_ into the biofuels, with higher photosynthetic efficiency and less cultivation space than higher plant [18].

#### Carotenoids

Carotenoids are lipophilic compounds and well known for the high biological activity. Most carotenoids have a C40 backbone structure and contain a long conjugated double bond, causing a greater ability to scavenge free radicals. Carotenoids are widely used in food and pharmaceutical industries for high biological potentials like anti-oxidant property, anti-tumor and anti-inflammatory effects [16]. Pharmacological and clinical studies have accepted the explanation that carotenoids induce the release of reactive oxygen species (ROS) from mitochondria, resulting in the cellular signaling of metabolic adaptation, differentiation and proliferation [19].

**Fig. 2:**
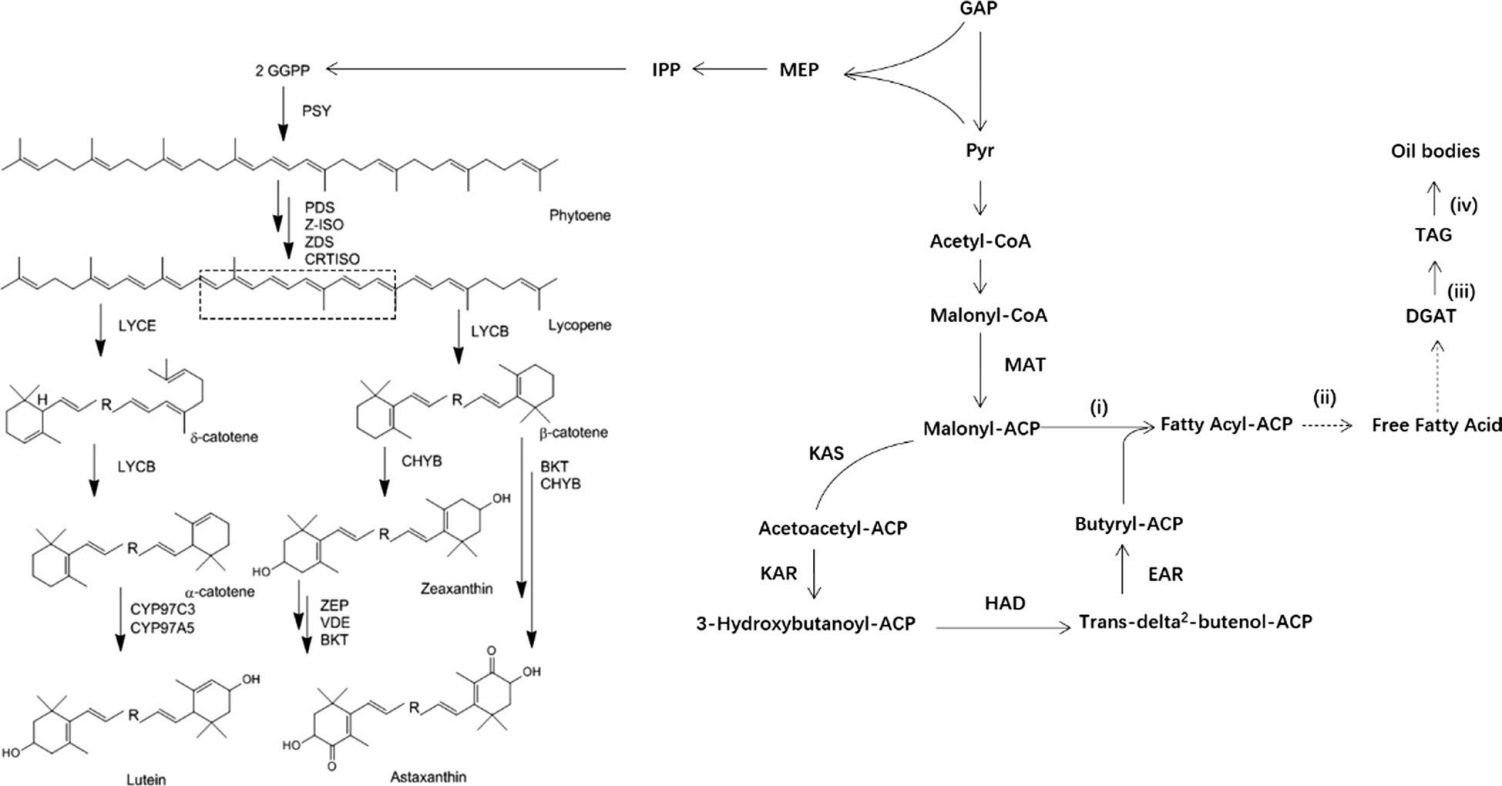
Biosynthesis pathways of carotenoids and TAG in plant and microalgae. The dashed box represents R in latter structural formula. The pathways of carotenoids involved several enzymes: *PSY* phytoene synthase, *PDS* phytoene desaturase, *Z-ISO ζ*-carotene isomerase, *ZDS ζ*-carotene desaturase, *CRTISO* carotenoid isomerase, *LCYB* lycopene *β*-cyclase, *LCYE* lycopene-*ε*-cyclase, *CHYB* carotene *β*-hydroxylase, *VDE* violaxanthin de-epoxidase, *BKT β*-carotene ketolase, *EMP* methylerythritol 4-phosphate, *IPP* isopentenyl pyrophosphate (adapted from [149]). The pathways of TAG involved several enzymes: *MAT* malonyl-CoA ACP transacylase, *KAS* beta-ketoacyl-ACP synthase, *KAR* beta-ketoacyl-ACP reductase, *HAD* beta-hydroxyacyl-ACP dehydrase, *EAR* enoyl-ACP reductase, *DGAT* diacylglycerol *O*-acyltransferase

As for the carotenoid biosynthesis, isopentenylpyrophosphate (IPP) and its allylic isomer dimethylallylpyrophosphate (DMAPP) are the building blocks in biosynthetic pathway of carotenoids, which are formed along with the non mevalonate pathway as from 3-phosphoglyceraldehyde (G3P) and pyruvate [20]. Then, the first C40 precursor phytoene is formed by condensation of two DMAPP by phytoene synthase (PSY) (Fig. 2). In most microalgae, the biosynthetic pathway of carotenoids to primary metabolite like lutein and secondary metabolite like astaxanthin is branched from lycopene by two types of cyclization reactions. Then highly diverse carotenoid family is produced by additional chain transformations such as hydroxylation and oxygen cleavage. *PSY PDS*, *LCYB, LCYE*, *CHYB* and *BKT* are regarded as key enzymes for the carotenoid biosynthesis (Fig. 2). Nitrogen starvation (N-starvation) is proved effective to upregulate the expression of *PSY* as well as other carotenogenetic genes like *PDS*, *LCYb*, *CHYb* and *BKT* with the increase of carotenoids [21].

#### Biodiesel production from lipids

The lipid yield from microalgae depends not only on biomass, but also on the intracellular oil content [22]. Biomass and lipid contend light and nutrients with different metabolic pathways. Generally, lipid content is accumulated in stressed environment like nitrogen starvation (N-starvation), sulfur starvation (S-starvation), phosphorus starvation (P-starvation) and high light intensity. N-starvation is regarded as the acceptable method to accumulate lipids [22]. Lipid accumulation triggered by S-starvation has been observed in several microalgae but poorly understood [23]. The redundant photons caused by high light can be dissipated as heat and fluorescence and are prone to induce the degradation of pigmentation and photosynthetic proteins, leading to attenuated photosynthetic capacity. However, since microalgae are prone to provide energy and precursors for cell division through catabolic processes, the lipid accumulation is limited for the cell cycle progression. The nutrient and culture conditions contribute different effects to lipid accumulation by activating the potential metabolic pathways like Kennedy pathway. In Kennedy pathway, triose phosphate is regarded as the primary precursor for triacylglycerol (TAG) (Fig. 2) [24]. According to the biosynthesis of each compound and the site, TAG biosynthesis is divided into four stages: (i) biosynthesis of free fatty acid in chloroplast, (ii) penetration of free fatty acid from chloroplast to cytosol, (iii) biosynthesis of TAG in endoplasmic reticulum and (iv) formation of oil bodies.

Transesterification of the lipids is the effective method to produce biodiesel from microalgae [18]. Many microalgae are able to produce intracellular oil to undergo stressed environment of nutrient starvation and high temperature, and can be converted to biodiesel by existing technologies, referred as transesterification. In this process, fatty acid methyl esters (FAMEs) or alcohol esters are formed by chemical catalytic reaction with organic solvent. New technology of “in situ” transesterification was proposed to eliminate extraction process by directly facilitating the formation of fatty acid ester [25]. Solvent, catalyst, temperature and operation conditions are the main factors to increase the extraction of production recovery and conservation of production quality [18].

#### Bioethanol from starch

Similar to producing biodiesel, microalgae appear to have several advantages to produce bioethanol like using less space. The cell structure of microalgae further enlarges the advantages. Microalgal cell wall differs from plant with low content of lignin or absence of lignin, which is beneficial for degradation of cellulose and hemicellulose [26]. In addition, the cell biomass provides necessary substrates of carbohydrate polymers (in forms of polysaccharide, starch and cellulose) for saccharification or degradation of bioethanol [27, 28].

To treat various microalgal feedstock composition, high-energy input like cell disruption and product extraction is needed to separate different cellular fractions and partly degrade carbohydrates, which subsequently can be used for bioethanol production by fermentation [28]. Before the fermentation, pretreatment and enzymatic conversions are common methods to realize saccharification in hydrolysis processes. Acid hydrolysis, enzymatic pretreatment and selection of hypercellulolytic mutants are efficient in production of bioethanol [27, 29]. For example, Harun et al. [30] had reported that acid pretreatment increased bioethanol yield by 2.25-fold and Choi et al. [31] had proved the high efficiency of enzymatic process in bioethanol. In these methods, temperature, enzyme and pressure are relatively significant factors to increase the production.

#### Feed supplements for aquaculture

Microalgae have the potential to replace the conventional raw materials in the aquaculture industry for their advantages of high protein and lipid contents [32]. The nutritional quality of microalgae has the positive effect on growth of aquatic species and the resistance to disease [33]. Although microalgae can grow in a wide range of habitats, their cultivation with aquatic species is still infeasible as they will compete with the dissolved oxygen, killing fish and other aquatic life. Therefore, microalgal cells can be harvested to feed fish. However, the commercial application of microalgae as the feed supplements is limited with the high cost of cultivation and the major application is focused as the feed ingredient for several specific beneficial properties, such as pigmentation [32, 34]. The pigment from microalgae can provide nutrient and bright color for the pet fish, as a means to increase the additional value in the aquaculture industry. As an alternative, the residual biomass from the cultivation process can be collected as feed supplements for the recyclable use. The functional proteins in microalgae are hardly extracted at a low cost; their retentions make the residual biomass as a plausible alternative to fishmeal protein [35]. The cell disruption during the extraction of products is also beneficial for the absorption of cell debris by fish and other aquatic life [28].

## Potential photosynthetic efficiency

Besides carbon metabolism, microalgal metabolism is accompanied by energy metabolism, like solar energy capture, delivery and dissipation, which play significant roles in carbon metabolism. The stoichiometric ratio of high-energy compounds, ATP and NAD(P)H influences redox state in microalgae, which is associated with carbon fixation, respiration and biosynthesis of carotenoids and TAG.

### Solar energy capture in microalgae

**Fig. 3:**
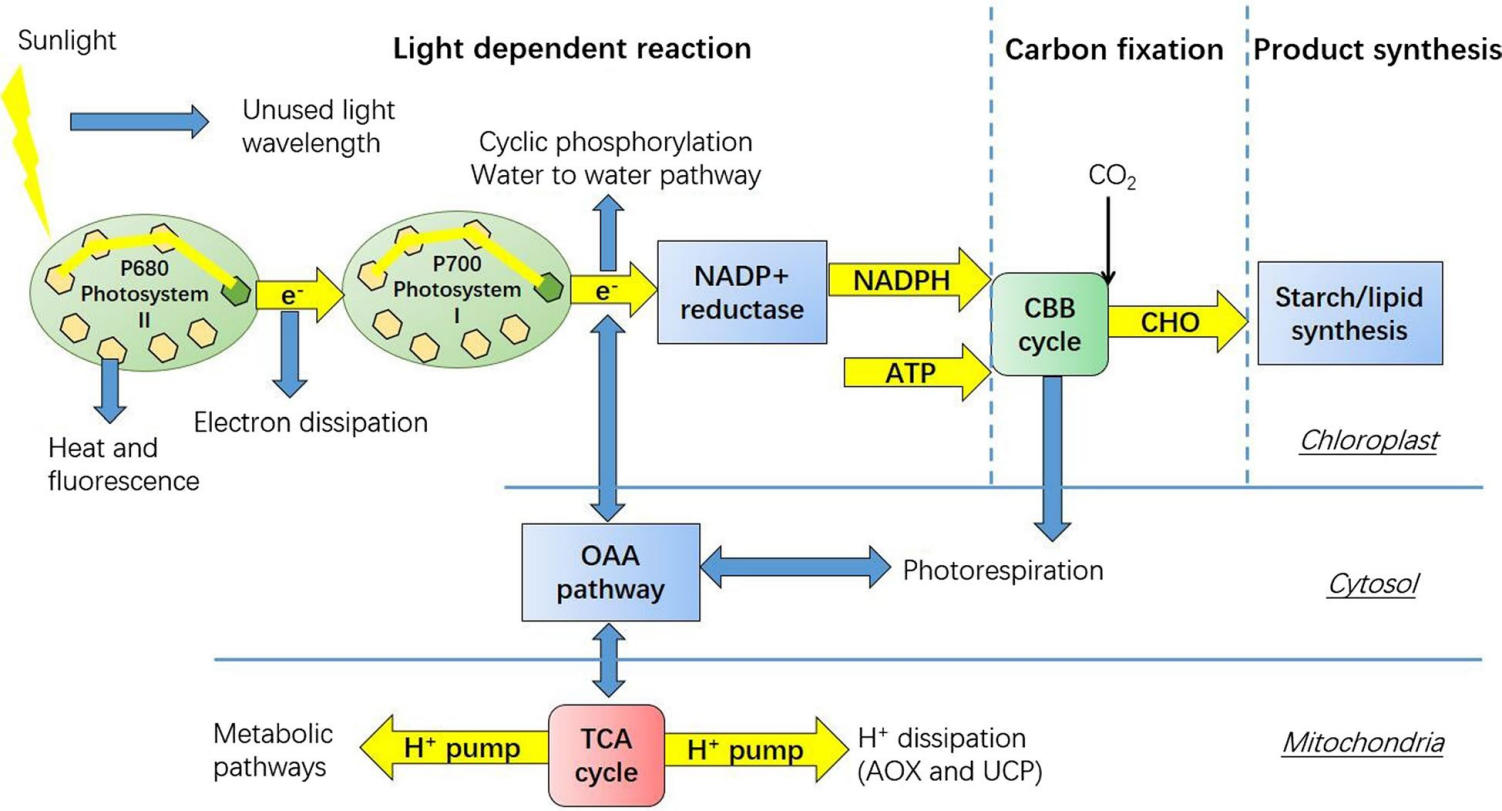
Energy capture, delivery and dissipation in plants and microalgae. When pigment molecules in Photosystem II absorb light, electrons are passed along an electron acceptor chain and finally to NADP^+^ reductase. The ATP and NADPH generated in light reaction are used to fix CO_2_. The intermediates from CBB cycle are then to participate in anabolism. The delivery of energy in microalgae depends on various pathways like malate/oxaloacetate shuttle and photorespiration. The energy dissipation depends on pathways like water to water pathway, photorespiration and H^+^ dissipation

Photosynthesis begins with the activation of assimilative pigment that is centered by light-harvesting antenna complexes (LHCs). Luminous energy is transported to chemical energy, which is determined by amounts and kinds of assimilative pigments as well as the state of restrict enzyme [36]. Minimum quantum of 8 is required for consumption of 2 NADPH molecules and 3 ATP molecules to assimilate 1 CO_2_ molecule in CBB cycle. Because of the weaker absorbance of chlorophyll in the green band, around 10% of the solar energy is intercepted as the absorbable light wavelength of microalgae is mainly in the range of 400–700 nm that contains 48.7% of the solar energy (Fig. 3) [37]. The re-emission of high-energy wavelength causes 6.6% loss of solar energy [38]. Meanwhile, delivery of energy between pigments and organs presents poor efficiency with 50–80% loss [6, 39]. The charge separation of complexes of PS I, Cytb6f, PS II, bounded to thylakoid membrane, limits the electron transport rate (1 ns, 10 ms, 1 μs), respectively [6]. The thermodynamics limit the amount of energy to do photosynthetic work with the 13.8% loss of the solar energy [37]. Therefore, it has potential to increase solar energy utilization in absorption and transfer of the energy of 21% increase, and transfer of electron of 9% increase [37, 40, 41].

### Solar energy delivery and dissipation in microalgae

During photosynthesis, chlorophyll traps energy from sunlight and produces ATP and NADPH to respond to the metabolic reactions. The energy is used to fix CO_2_ for the biosynthesis of organic carbon-based compounds [42]. There are two types of electron flow present in light-dependent reaction: linear electron flow (LEF) and cyclic electron flow (CEF). LEF produces two ATP molecules and one NADPH molecule for every two electrons that enter the pathway, while CEF produces ATP for two electrons. In addition, water to water pathway (electron flow from PS II to PS I and then form H_2_O) has been paid much attention due to the absence of NADPH in this pathway [43]. The plant and microalgae are believed to reroute part of the electrons to alternative sinks other than NADP^+^, like O_2_, NO_2_^−^ [6].

Cofactors like ATP and NAD(P)H play important roles in regulating intracellular environment to orientate the metabolic pathways into biomass accumulation or product biosynthesis in the various cultivation conditions. However, ATP/ADT and NAD(P)H/NAD(P) are shared in numerous pathways and protein transports. The translocators located in membranes are biosynthesized by enough ATP amount, and it has been reported ATP directly participates into about 200 metabolic pathways [44]. NAD(P)H/NAD(P) couples diffuse electrons to the targeted products and also generate a large amount of ATP. NADPH is usually activated in CBB cycle to fix CO_2_. Given that the stoichiometric ratio of ATP and NADPH per CO_2_ fixed is 3:2 and the ratio from LEF is merely 2.6:2; the ATP is deficient in CO_2_ fixation. As a result, the reducing equivalents are accumulated in the chloroplast, which may result in the expression of antioxidant and defense genes. Chloroplast is evolved to counteract the imbalance by regulating several important biological processes. Under high light conditions, changes of plastoquinone (PQ) pool are known to balance and maintain photosynthetic energy distribution between PSII and PSI in the short term and photosystem stoichiometry such as light-harvesting proteins is adjusted to counteract the imbalance in the long term [34].

A tight stoichiometric ratio of ATP and NADPH in chloroplast is needed to fix CO_2_, while the excess NADPH produced from CEF will be accumulated in chloroplast or transferred into cytoplasm and mitochondria, which affects the cellular redox state, and finally leads to the increase of ROS [19]. The mitochondrial oxidative metabolism has been emphasized as a promising orientation for maintain high photosynthetic efficiency [8, 45]. The metabolism functions to dissipate energy as heat or ROS at expense of energy generated in intracellular environment, which is regarded as the regulator of mitochondrial redox state and ROS generation. In addition, cytochrome oxidase (COX) is limited by ratio of ATP/ADP under excess NADPH condition; consequently, the ability to consume NADH is restricted [45]. As an alternative, the non-phosphorylation of alternative oxidase (AOX) can play the oxidization that is unaffected by the ratio [8].

Generally, photosynthesis and respiratory processes are energy-generating processes that are involved in redox regulation and ROS metabolism. The processes are interlinked and influence the energy flux and redox fluctuations between chloroplast, mitochondria and cytosol. A high level of metabolic coordination and balance between signaling and metabolic pathways is required to maintain energy flux through organelles with less ROS produced. Recent studies link rate of ascorbate biosynthesis with the two processes [34]. The ascorbate–glutathione cycle enzymes such as glutathione reductase and glutathione peroxidase are revealed to be co-expressed in chloroplast and mitochondria, which are considered as redox state reporters to reflect divisions between energy generated and used. Ascorbate is an important antioxidant in various metabolic pathways that contributes to the cellular redox state such as photoprotection [34]. The formation of ascorbate is attributed to the alternative electron donor of l-galactono-1,4-lactone dehydrogenase in photorespiration. Therefore, the photosynthetic efficiency can be enhanced by increasing the ascorbate even though the TCA cycle is impaired. The regulation shows an ascorbate-mediated manner in photosynthesis and respiratory. In addition, another mechanism has also been proposed to reinforce the high degree of metabolic co-ordination. For example, uncoupling proteins located in inner mitochondrial membrane are related to maintain the redox poise of mitochondrial electron transport chain, especially when the demand of oxidation of NADH is high, showing potential to facilitate both TCA cycle and photosynthetic metabolism [46].

Energy dissipation is usually ignored in the microalgal researches that cause nutrient loss in the cultivation processes. During photosynthesis, chlorophyll and carotenoid molecules located in LHCs are able to efficiently absorb the light and transfer the energy into RC. However, part of the energy will be dissipated by carotenoid as heat [47]. As shown in Fig. 3, light is captured by chlorophyll and forms the excited state in PSII, then the energy is transferred to carotenoid [48]. The carotenoid in excited state is unstable and then dissipates the energy to form ground state. This regulatory process maintains the balance between dissipation and utilization of solar energy to minimize generation of oxidizing molecules and thus protect microalgae from the excess photons. Subsequently, electrons from water in PSII are transferred to PSI to generate NADPH; otherwise, they dissipate by water to water pathway or cyclic phosphorylation. Water to water pathway reroutes electron to generate H_2_O_2_, without formation of energy. In this pathway, electron from PSII is used to reduce atmospheric O_2_, thereby generate O_2_^−^. Then the O_2_^−^ is reduced to H_2_O by the catalyzations of superoxide dismutase and ascorbate peroxidase. This pathway provides a “safe” way to dissipate excess photons with high electron flux. During photorespiration, malate/oxaloacetate shuttle and other pathways of reducing power exchange are performed to deliver NADPH/NADH among chloroplast, cytosol and mitochondria to generate H_2_O_2_ in cytosol, which can be catalyzed by peroxidase to generate H_2_O.

### Energy consumption in microalgae

The metabolism of microalgae is associated with the energy consumption, such as CBB cycle and TAG biosynthesis. Evaluating theoretical energy consumption in microalgae is essential for enhancing the biomass and product yields by rerouting the energy in metabolic flux. Polysaccharides are typically used as the energy and carbon reserves. In microalgae, starch is the most common type of polysaccharides that stores carbon and energy as the primary metabolite. Other polysaccharides in microalgae are widely distributed in intracellular environment like chrysolaminarin [49] and extracellular environment like exopolysaccharides [50]. The biosynthesis of intermediates like proteins and sugars is responsible for cell growth, like forming cytoskeleton to enlarge the cell size and guiding the phragmoplast to realize cell division [46]. The energy for biosynthesis of 1 molecule C_6_-sugars requires 18 ATP molecules and 12 NADPH molecules, and the energy to link sugars requires 1 ATP/sugar. Normally, it requires 8 photons to fix 1 CO_2_ molecule, which actually requires 3 ATP molecules and 2 NADPH molecules. Therefore, the lowest photon consumption on acetyl-CoA is 16 mol mol^−1^ and on C6-sugars is 48 mol mol^−1^, respectively. To evaluate the efficiency of photon utilization, index of *Y*_*P*_ is used and described as follows:

1 $$Y_{P} = \frac{{{\text{Product }}\,{\text{biosynthesized}}}}{{{\text{Protons abosorbed}} }}$$

and the maximum *Y*_*P*_ for acetyl-CoA is 6.25 × 10^−2^ and for starch is 2.08 × 10^−2^ mol mol^−1^, respectively.

The lipid usually represents more concentrated storage of energy. In lipid biosynthesis, the requirement of energy is in high level [44], the formation of C18:0 requires 1 acetyl-CoA, 8 malonyl-CoA and 16 NADPH. In this situation, extra energy from intracellular environment is imperative. For example, the proteins are degraded to provide energy as the lipid content increases. In the nutrient-limiting conditions, the rate of product biosynthesis reaches the highest at the initial hours, then gradually decreases after that. The process of product biosynthesis is maintained by supplying energy like excess light (EL) [51]. To optimize environmental conditions for lipid biosynthesis, the maximum productivity is required to be revealed. Guiding 1 molecule CO_2_ in starch requires 3.17 ATP molecules and 2 NAD(P)H molecules, which is equal to 8.17 ATP molecules, and 1 molecule CO_2_ in TAG requires 2.30 ATP molecules and 2.81 NAD(P)H molecules, equal to 9.32 ATP molecules [6]. The lowest photon consumption on TAG (C_53_H_94_O_6_) is 592 mol mol^−1^. Therefore, the maximum *Y*_*P*_ is 1.69 × 10^−3^ mol mol^−1^ for TAG (C_53_H_94_O_6_). The need of energy for TAG is higher than starch.

## Approaches for enhancing biomass and product accumulation mainly through the efficient carbon partitioning

Advances of methods based on microbial physiology in biomass accumulation of microalgae have been untied with gene technology and fermentation model to increase the valuable products or save the industrial cost. The designs of cultivation modes and methods to maintain energy (primarily from light) and nutrient balances have the objective of creating a suitable environment for cell growth and the designs to disturb the balances aim at forming the stressed environment for product biosynthesis (Table 1).

**Table 1:** Effects of nutrient and environmental conditions on bioproduct production

Stress	Species	Products	Biomass titer		References
			Biomass yield (mg L^−1^ h^−1^)	Product content (mg g^−1^)	
Nutrient and environmental conditions on biomass accumulation					
Glucose	*C. zofingiensis*	Astaxanthin	292.8	0.8–1.0	[52]
CO_2_	*C. vulgaris*	Biomass	9.2	–	[53]
Light	*Scenedesmus almeriensis*	Lutein	36.2	5.5	[54]
Temperature	*C. vulgaris*	Biomass	9.2	–	[53]
pH	*Scenedesmus* sp.	Lipid	23.6	400.0–450.0	[55]
Nutrient and environmental stresses on bioproduct production					
Nitrogen	*C. vulgaris*	TAG	9.0	617.5	[56]
	*S. obliquus*	Lipid	9.0–18.3	91.7–117.1	[57]
Phosphorus	*S. obliquus*	Lipid	–	530.0	[58]
Ca	*C. vulgaris* and *S. obliquus*	Lipid	1.6–2.5	100.0–400.0	[59]
Mg	*C. vulgaris* and *S. obliquus*	Lipid	1.8–3.0	100.0–260.0	[59]
Light	*H. pluviali*s	Astaxanthin	8.2	51.9	[34]
Temperature	*Monoraphidium* sp. SB2	Lipid	3.9	329.0	[60]
Salinity	*C. vulgaris* and *S. obliquus*	Lipid	2.3–1.0	170.0–400.0	[59]
pH	*Chlorella* CHLOR1	Triglyceride	–	–	[61]

### Optimizing growth conditions

Conditions of microalgae are key factors to increase biomass and the product yields. However, the conditions significantly differ from various microalgae used. Therefore, we discuss these conditions in aspect of methods to improve environment, and thus the methods can be applied in various microalgae.

#### Light

Light is the critical factor to increase biomass accumulation and product biosynthesis. In mass cultivation, enhancing light intensity is the traditional way to provide sufficient protons to increase biomass, while the effect is unsatisfactory due to the photoinhibition as cell number increases. Furthermore, a considerable amount of heat will be generated if the light contacts closely the microalgae. As an alternative, innovative light source, which possesses a narrow spectral output to overlap the photosynthetic absorption spectrum of microalgae used, is needed to increase the conversation of light energy and decrease the elimination of heat. Therefore, searching the optimal light wavelength with LED light is the other way to increase biomass with high photosynthetic efficiency, which is also the active field in past several years [62, 63]. However, although the application of blue light is efficient to increase cell size and the red light is favorable for high division rate [64], the application of the single light is still challenged with low cell activity, which is mainly due to the fact other light wavelengths are also essential for the cellular metabolism. Therefore, mix of red and white light at 1:1 was used to increase the cell activity and maintain the activity. The desired mixing is to induce cell to be active with the highest photosynthetic efficiency [39].

The industrial performance of microalgae by autotrophic cultivation is limited by cell concentration and light supply. Open ponds applying natural light is common option for mass cultivation, while the biomass is merely around 0.5 g L^−1^ [65]. Therefore, various designs of PBR have proposed to increase utilization of light. The tubular bioreactor increases average biomass to 5 g L^−1^ than open raceway pond [65]. Sato et al. [66] established a vertical column bioreactor for *Fistulifera* sp. with specific growth rate of 0.29–0.57 g L^−1^ and Araya et al. [67] used panel bioreactor for *Chlorella vulgaris* with specific growth rate of 0.131 g L^−1^ day^− 1^.

#### Nutrients

Under suitable environmental conditions, the sufficient nutrients lengthen the steady-state growth of microalgae with high growth rate [14]. However, the initial nutrient concentrations are limited by the microalgae used. Consequently, in most cases, the concentrations are insufficient to make microalgae grow in steady-state for a long time. The extra operation is promising to avoid the decline of cell activity [34, 68]. Relationship of substrate, biomass, product and fed-batch concentration is described as Eq. (2) where *C*_*s*_, *C*_*x*_ and *C*_*p*_ are the concentration of substrate, biomass and products, *C*_*F·s*_ is the feeding concentration of substrate, *Y*_xs_ and *Y*_ps_ are growth yield on nutrients and products, *μ*_*sx*_ is the specific consumption rate of nutrients, *n* is product content and *t* is the cultivation time. The stable nutrient concentration in culture process is the most important factor to keep the growth rate in a high level and to achieve high biomass. Adding nutrients is usually done during cultivation. To avoid the effect of dilution rate, fed-batch culture is feasible to improve biomass.

Three difficulties are needed to overcome in fed-batch: the feeding time, feeding amount and ratio of different nutrients in fed-batch media, which direct various types of fed-batch culture (Table 2). The ratio of different nutrients in fed-batch culture is essential. The biomass on nutrient concentration (*Y*_xs_) is stable to specific strain, which determines the ratio in fed-batch media. Nutrient-fold fed-batch culture is unsuitable because *Y*_xs_ of nutrients differs from the initial ratio of nutrient concentration. PH-stable fed-batch culture is proposed to solve feeding time, while the amount is hard to control. Exponential fed-batch culture can be used to effectively overcome the difficulty. As shown in Eq. (2), the nutrient concentration is maintained constantly when d*C*_*s*_/d*t *= 0. However, in high microalgae cell density, the changing environment requires more energy to maintain, which may influence the effect of fed-batch.

**Table 2:** Types of fed-batch to increase biomass

Types of fed-batch culture	Species	Enhancement (B: biomass concentration, S: specific growth rate)	References
Nutrient-fold fed-batch culture	*C. sorokiniana*	B:176.1%	[69]
pH-stable fed-batch culture	*H. pluvialis*	S: 20.8%	[39]
Exponential fed-batch culture	*H. pluvialis*	B: 93.9%	[34]
Replacement-nutrient of fed-batch culture	*H. pluvialis*	–	[70]
Membrane filtration	*Chlorella* sp.	B: 687.4%	[71]
Periodically harvesting	*C. vulgaris*	B: 46.48%	[72]

2 $$\frac{{{\text{d}}C_{s} }}{{{\text{d}}t}} = C_{F \cdot s} - \frac{1}{Yxs}\frac{{{\text{d}}C_{x} }}{{{\text{d}}t}} - \frac{1}{{Y_{ps} }}\frac{{{\text{d}}C_{p} }}{{{\text{d}}t}} - \mu_{\text{sx}} C_{x}$$

3 $$C_{F \cdot s} = \frac{1}{{Y_{\text{xs}} }}\mu_{x} C_{x} + \frac{1}{{Y_{\text{ps}} }}\mu_{{_{p} }} C_{{_{p} }} + \mu_{\text{sx}} C_{x}$$

4 $$C_{p} = n\frac{{Y_{\text{ps}} }}{{Y_{\text{xs}} }}C_{x}$$

5 $$\mu_{p} = n\frac{{Y_{\text{ps}} }}{{Y_{\text{xs}} }}\mu_{x}$$

6 $$C_{F \cdot s} = \left( {\frac{1}{{Y_{\text{xs}} }}\mu_{x} + \frac{{Y_{\text{ps}} }}{{Y_{\text{xs}}^{{^{2} }} }}n^{{^{2} }} \mu_{x} } \right)C_{0} \,{\text{exp (}}\mu t )$$

#### Temperature

The absorption of nutrient is also influenced by changes of the temperature, which is mainly through regulation of the enzymes involved in cell growth [73]. Normally, the cell growth rate is enhancing as the temperature increases to a threshold value that is optimal for cell growth, and then decreases as the temperature exceeds this value. The traditional temperature for most microalgal growth is considered to rang from 14 to 35 °C [73]. The optimal temperature for cell growth is largely dependent on the species used. A comparative study involving in effect of temperature on microalgal growth suggested the maximum biomass concentrations were obtained at 14 °C for *Nannochloropsis oculata*, at 26 °C for *Dixioniella grisea*, at 14 °C for *Rhodomonas salina* and 14 °C for *Isochrysis galbana* after 14 days of cultivation [74]. Even though the species are same in the cultivation process, the optimal temperature is still influenced by other environmental conditions. For example, the optimal temperature for *C. vulgaris* growth ranged from 25 to 30 °C as the previous researches reported [53]. Therefore, it was of significance to optimize the temperature condition in microalgae cultivation.

#### pH

PH is considered as the critical environmental factor that controls cell metabolism and accumulation of biomass. It seems all the microalgae strains have a small optimal range of pH for high cell growth rate. The growth of *C. vulgaris* at acidic (3.0–6.2), neutral (7.5–8.0) and alkaline (8.3–9.0) pH suggested neutral pH resulted in the maximum cell growth rate [75]. The similar neutral pH range was also reported to be suitable for *Nannochloropsis salina*, *Chlamydomonas applanata* and *Dunaliella bardawil* [76–78]. In addition, a series of experiments revealed alkaline pH was at the optimum range for the growth of *Chlorella ellipsoidea*, *Dunaliella salina* and *Nannochloropsis salina* [78, 79]. Although the acidic pH is unsuitable for most microalgae strains, the pH still has the strong effect to promote cell growth of *Euglena mutabilis* [73]. The environmental pH is known to be capable of influencing the absorption of nutrient and photosynthetic efficiency; its optimization is beneficial for the increase of biomass concentration.

### Maximizing product

The products from microalgae can be categorized into primary metabolites such as H_2_, lutein and secondary metabolites like astaxanthin, β-carotene [16]. High temperature, high light intensity, suitable light wavelength and nutrient-limitation are frequently used conditions to promote product biosynthesis (Table 1). Most of the conditions result in the elevated carbon availability, which is considered as the key factor to increase the product biosynthesis. However, these conditions are usually different to the conditions for biomass accumulation.

#### Nutrients

Nutrient-limitation is the conventional stress-based strategy to increase the product yields [56, 80]. As the nutrients such as nitrogen, sulfur and phosphorus are important elements for the biosynthesis of protein, lipid and other central intermediates, their deficiency in media can increase intracellular carbon availability by the degrading of the macromolecular substances, which also leads to growth retardation or even cell death. Nitrogen starvation (N-starvation) is widely used strategy to increase starch and lipid accumulation [56]. Once responding to N-starvation, microalgae redirect the carbon skeletons from proteins into the central carbon-metabolism intermediate of pyruvate, which could be used to synthesize starch and lipid as the storage forms. Since microalgae require lower energy to guide *per* carbon molecule into starch than lipid, they would prefer to guide the carbon molecules into starch. Therefore, N-starvation is effective to induce carbon into lipid, while the conversion efficiency is limited by the energy requirement. Phosphorus starvation (P-starvation) is known to increase the TAG levels, potentially by phospholipid degradation. Kamalanathan et al. [81] reported P-starvation promoted at least 2.4-fold higher per cell than nutrient-replete conditions in *C. reinhardtii*. In addition, several researches also indicated P-starvation resulted in higher cellular lipid levels [58, 82]. The literatures on other element metabolisms such as sulfur metabolism are rather limited.

#### Temperature

High temperature has strong effect on cell activity, which influences the enzymes in product biosynthesis. Under temperature at 30 °C, 7.4% increase of lipid productivity was achieved in *Monoraphidium* sp. SB2 [60]. The effect of temperature on product biosynthesis is different due to the microalgae used. The reported researches have shown that temperature at 35 or 30 °C is prone to induce astaxanthin accumulation in *H. pluvialis* [83, 84], while others have proved lower temperature at 25–30 °C is preferable [85]. The synergistic effects of temperature and other factors have strong enhanced effect on product biosynthesis. Combing temperature and light intensity significantly increases TAG content and productivity in *Isochrysis galbana* [86]. In addition, low sub-optimal temperatures combined with exogenous glycine betaine at 500 mg L^−1^ improved the lipid productivity [87].

#### Light

Product biosynthesis of microalgae is an energy-demand process with high level of reactive oxygen species (ROS). High light intensity provides both of them for microalgae. Under high light, cellular morphology changes with less activity, the cell division slows down and cell wall becomes hard and thick. An increase of lipid content in *Scenedesmus abundans* is realized under higher light with 20.9% increase to proximate result [88]. Neutral lipid content of more than threefold increase is observed in *Chlorella* sp. and *Monoraphidium* sp. at the expense of degradation of chlorophyll and reduction of protein, carbohydrate content and membrane lipid [89]. The light quality is also important for product biosynthesis. The blue light has the potential to induce microalgae cell to synthesize product and accelerate this process. Atta et al. [90] has obtained maximum lipid content (23.5%) due to high efficiency and deep penetration of 200 μmol m^−2^ s^−1^ of blue light within reduced cultivation time of 8 days. Mixed light of blue and white increases 11.8% of astaxanthin in *Haematococcus pluvialis* [34]. Sufficient work has been done on light-induction to maximize the product.

#### Others

**Fig. 4:**
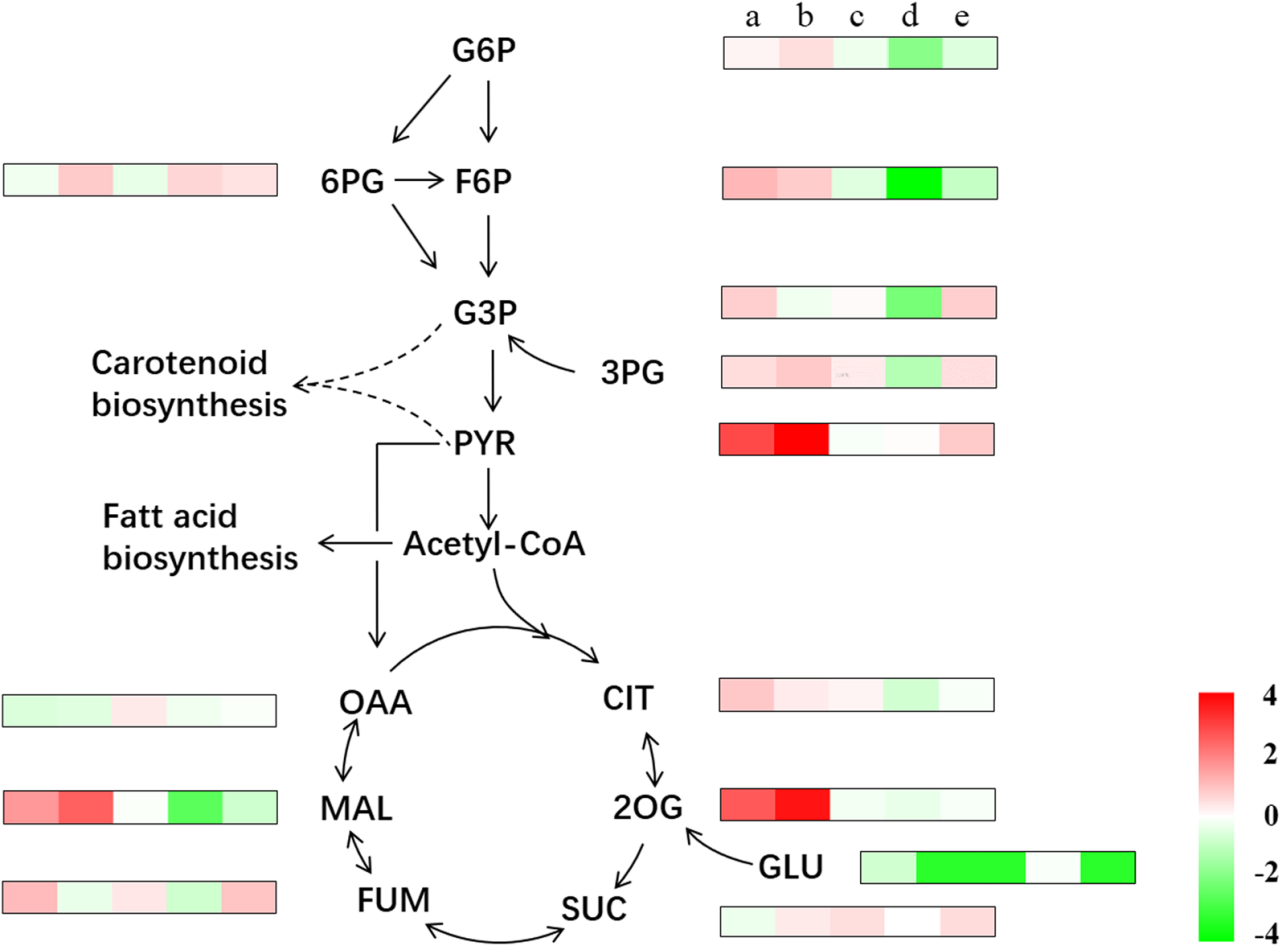
A comparison of fold changes of the metabolites in molecular level associated with the stressed conditions. The boxes show the log twofold changes and the letters of a, b, c, d and e above the boxes represent the comparisons of +N+EL/+N, −N/+N, −N+Ses/−N, +N+DCMU/+N and −N+EL/−N, respectively. The carbon flux can be regulated by N-starvation and EL. The carbon metabolites: *G6P* glucose-6-phosphate, *F6P* fructose-6-phosphate, *6PG* 6-Phosphogluconic acid, *G3P* glyceraldehyde-3-phosphate, *3PG* 3-phosphoglycerate, *PYR* pyruvate, *OAA* oxaloacetate, *MAL* malate, *FUM* fumarate, *SUC* succinate, *2OG* 2-oxoglutarate, *CIT* citrate, *GLU* glutamate (data from [93])

Environmental factors like salts, pH and ROS also affect product biosynthesis. Salts in environment influence osmotic pressure of cell. Pelah et al. [91] found under the treatment of 2% sodium chloride that total carotenoids increased by 16.7% in *Chlorella zofingiensis*. When the pH was adjusted to 8 in *Scenedesmus abundans,* 19.3% increase of lipid productivity to proximate result was obtained [88]. High ROS level was proved to correlate well with astaxanthin content in *C. zofingiensis* [92]. The high ratio of C/N at 11.2 produced 3.7-fold astaxanthin coupled with salinity stress [85]. Our previous findings indicated a competitive relationship between the biosynthetic pathways of starch and lipid under stresses and the elevated carbon skeleton was redirected into starch under EL and N-starvation (Fig. 4) [93]. The additional EL was prone to accelerate the degradation of protein with 1.68-fold increase of PYR, which means more carbon molecules were induced into process carbon partitioning. Interestingly, G6P and F6P contents decreased after additional treatment of EL, with the 1.62-fold increase of G3P, which suggested EL was capable to reroute carbon flux into carotenoid and lipid. In addition, the combined effect of EL and N-starvation redirected the organic carbon from storage polysaccharide toward carbon catabolism by glycolysis, PPP and TCA cycle. The effect was possibly dominated by intracellular ROS and excess photons. The reducing equivalents produced by excess photons appeared to activate the starch degradation. G6P and F6P decreased as the level of intracellular ROS was reduced by sesamol, which suggests the increased ROS promoted starch biosynthesis. However, the higher level of intracellular ROS induced by EL and N-starvation limited the precursors of polysaccharide and enhanced lipid accumulation. The finding that *C. reinhardtii* converted starch into lipid was also consistent with reported researches [94–96], which might suggest that starch served as the major carbon source for lipid production. Consequently, the carbon partitioning was first oriented to starch as the stored substance in response to N-starvation. However, as the stressed level increased, the microalgae chose to convert starch into lipid. Therefore, impairing the starch biosynthesis might impair the lipid biosynthesis.

Although the stress-based strategies promote the biosynthesis of products, the biomass concentration is limited by the conditions. To maximize the productivity or reduce the cost, more efficient approaches such as multi-stage cultivation and bacteria–microalgae co-cultivation are used by combined the stresses [97].

## Key engineering strategies

Microalgae are regarded as promising cell factories for metabolic engineering. In recent years, the release of whole genome sequence of many microalgae, such as *C. vulgaris, Nannochloropsis, Phaeodactylum tricornutum* [80], benefits the metabolic pathway regulation and genetic engineering. In most researches, metabolic engineering aims at guiding carbon and energy fluxes to targeted pathway or products, while the synergistic effect of the two fluxes is seldom concerned.

### Carbon allocating into biomass and product

As mentioned above, microalgae orient 5–10% carbon partitioning into several fatty acids and terpenoids, which are required in steady-state growth. The CBB cycle and TCA cycle are linked to carbon source of CO_2_ and glucose in carbon flux and also linked to numerous metabolic pathways like PP pathway; thus the two cycles show significant roles in metabolism of microalgae. Engineering pathways of carotenoid and lipid biosynthesis are the active fields as their pharmaceutical and food functions are being revealed and the benefits of biodiesel from microalgae also attract many researches (Table 3).

**Table 3:** Carbon allocating in carbon fixation and lipid biosynthesis by metabolic engineering

	Species	Carbon source	Product	Results	Strategy	References
Light-dependent reaction	*C. reinhardtii*	CO_2_	Biomass	Solar conversion efficiencies and photosynthetic productivity are enhanced	Reducing antenna size	[98]
	*Anabeana simensis*	CO_2_	Biomass	Solar conversion efficiencies, photosynthetic productivity and growth rate are effected	Changing pigment composition	[99]
CO_2_ fixation	–	CO_2_	Biomass	Solar conversion efficiencies are enhanced	Reducing Rubisco oxygen affinity or increasing Rubisco catalytic rate	[100]
	*Synechocystis* 6803	CO_2_	Ethylene	10% of fixed carbon is diverted into ethylene and photosynthetic activities is increased	Engineering TCA cycle	[7]
	*Synechocystis* 6803	CO_2_	Acetate	More than 20% of carbon is channeled into the triose sink	Engineering PP pathway	[101]
Product biosynthesis	*C. reinhardtii*	CO_2_	Carotenoid	2.0- and 2.2-fold of carotenoids violaxanthin and lutein are increased	Overexpressing *PSY*	[102]
	*Thalassiosira pseudonana*	Protein, carbohydrate	Lipid	3.3-fold increase of lipids	Engineering a multifunctional lipase/phospholipase/acyltransferase	[103]

#### Engineering CBB cycle

CBB cycle is a redox-regulated process. Reversible redox post-translational modifications play an important role in regulation of cell metabolism by transforming cysteine residues to different forms, which have been recognized early to regulate the enzymes in CBB cycle. In addition, most of the enzyme reactions are controlled by thioredoxins (*TRXs*). In CBB cycle, 15 enzymes such as of *RPE, RPI, FBA, TPI, PGK* are proved to be activated by *TRXs* [104]. Meanwhile, Rubisco activase (RCA) and *CP12* are also regulated by *TRXs*.

*TRXs* are reduced in photosynthetic electron transfer chain by the oxidization of ferredoxin/thioredoxin reductase, which means *TRXs* are the connections between chloroplast and mitochondria by electron transfer of NAD(H)/NADP(H). The coding genes of *gapA* and *gapB* of photosynthetic glyceraldehyde-3-phosphate dehydrogenase are effected by *TRXs* in different levels; the former one is inactive to *TRXs*, which requires the cooperative action of *CP12*. Therefore, CBB can be effectively regulated by *CP12* via NAD(H)/NADP(H) ratio. Tamoi et al. [105] observed that growth rate of ScΔCP12 (*Sccp12*-disruption) mutant cells was significantly slower than wild-type cells and O_2_ consumption rate was 0.43-fold lower than wild strain under dark conditions and therefore concluded that the oligomerization of *CP12* regulated the carbon flux from CBB cycle to PP pathway. RCA acts on Rubisco by cutting off the inactive ribulose bisphosphate by ATP-dependent reaction and then the free Rubisco is formylated by combination of Mg^2+^ and CO_2_. Therefore, RCA directly effects Rubisco activity and CO_2_ fixation. The gene *CBBX* had been originally recognized in the function of carbon fixation. The *CBBX* is unclear and the functional *cbbX* genes appear to be associated with red-type Rubiscos [106]. Meanwhile, hypothesis of redox regulation of activase, by oxidizing or forming a disulphide bond in C-cysteines extension of the α–isoform, is used to regulate RCA. To effectively reply to non-steady state photosynthesis, enhancing RCA thermostability and light tolerance is also essential [107, 108].

#### Engineering TCA cycle

The TCA cycle is activated as two architectures depend on whether the terminal electron acceptor is available in the reaction. TCA cycle can be divided into the reductive branch from oxaloacetate to succinyl-CoA and the oxidative branch from citrate to 2-oxoglutarate. When the NAD^+^ is available for electron, TCA cycle acts to oxidize substrate to CO_2_ and produce NADH. Otherwise, TCA cycle acts to produce intermediates for anabolism such as citric acid, l-glutamic acid. However, the intermediate yield in TCA cycle is analyzed to be lower than the theoretical maximum yield (*Y*^*E*^) [9]. The turnover rate of carbon in TCA cycle is limited [7].

Engineering TCA cycle is prone to allocate carbon in targeted products at the expense of the generation of energy. Therefore, pathway of intermediate biosynthesis that requires low level of energy is discussed to alleviate the undesirable effect of energy [9]. Replacing electron acceptor of NAD^+^ to decrease the gibbs free energy of the reaction might induce the reaction to occur more easily [109]. Glyoxylate shunt in TCA cycle can produce succinate without input of energy and construct bridge between the reductive branch and oxidative branch. In microalgae, upregulating glyoxylate shunt can increase the turnover of carbon in both TCA and CBB cycle and thus the efficiency of carbon fixation is enhanced [7].

#### Engineering pathways of carotenoid and lipid biosynthesis

The key metabolic steps and crucial genes that control biosynthesis of algal carotenogenesis have been reviewed and applied in many researches [110]. The genes of *PSY*, *PDS*, *LCYb*, *CHYb* and *BKT* are suggested as crucial hinges during the carotenogenesis [111–113]. *PSY* is the rate-limiting enzyme in the pathway of carotenoid biosynthesis and the coding genes of *PSY* is different in plants and microalgae [21, 114]. Overexpressing *PSY* in *C. reinhardtii* by nuclear transformation increases 2.0- and 2.2-fold of carotenoids violaxanthin and lutein to untransformed cells [102]. The *PDS* gene mutation is a promising approach to increase astaxanthin in *H. pluvialis* [115]. In the carotenoid biosynthesis, lycopene can be converted to δ-carotene (α-branch) by *LCYE* and γ-carotene (β-branch) by *LCYB* [116]. Several microalgae such as *H. pluvialis* and *C. zofingiensis* can synthesize astaxanthin from β-carotene by the action *BKT*. The transgenic *Arabidopsis thaliana* with *BKT* from *C. reinhardtii* (*CrBKT*), *C. zofingiensis* (*CzBKT*) and *H. pluvialis* (*HpBKT3)* has different development in increasing astaxanthin in orange leaves. Overexpressing *CrBKT* is capable of increasing 1.8-fold in total carotenoids, while the operation of *CzBKT* and *HpBKT3* has no influence on carotenoid accumulation [117]. Furthermore, the carotenoid biosynthesis is a complex multi-enzyme pathway; the effect of expression of a certain gene controlling the pathway in carotenoid biosynthesis might depend on the level of other genes. Therefore, the transcription factors like phytochrome-interacting factor can activate multiple components in the pathway to increase the products [118].

Engineering lipid pathway is an efficient way to increase lipid content in microalgae. Several studies have explored the overexpression of genes to increase lipid product in lipid pathway. In Kennedy pathway, TAG biosynthesis is divided into four stages. The pathway for acetyl-CoA undergoes carboxylation by acetyl-CoA carboxylase (ACCase), which is a limiting enzyme in lipid pathway; (i) Roesseler et al. [119] found the level of ACCase increased to two- to fourfold in the silicon deficiency environment, which promoted the lipid biosynthesis. Consequently, many efforts have been focused on overexpression of ACCase, while the results are unsatisfactory. Dunahay et al. [120] increased ACCase level by engineering ACCase genes from *Cyclotella cryptica* T13L into *Cyclotella cryptica* CYCLO1 and *Navicula saprophila* NAVIC1, while the lipid content was changeless. To overcome the limit of ACCase, a metabolic sink is required for overproducing the free fatty acid. The sink provided by expression of thioesterase activity has the potential to accelerate metabolic flux from the complex fatty acids to free fatty acids. (ii) For example, Davis et al. [121] induced the ACCase overexpression with thioesterase and achieved a sixfold increase in the rate of fatty acid synthesis. In addition, overexpression of thioesterase merely has been proved to successfully increase the lipid productivity [122]. Diacylglycerol acyltransferase is responsible for the last step from DGAT to TAG. (iii) The overexpression of the coding genes such as *AtDGAT1* and *SiDGAT1* is effective to increase oil content [123, 124]. During the lipid droplet formation, particular proteins localized on the surface perform to prevent fusion of lipid droplets and act as enzymatic role. (iv) N-starvation can be applied in identification of lipid droplet protein by proteomic analyses [125]. In summary, the pathway of lipid biosynthesis is still unclear to us.

### Energy rerouting in chloroplast and mitochondria

Simply, carbon fixation is a ATP-demand process and product biosynthesis is associated with NAD(P)H. Therefore, engineering cofactors of ATP and NAD(P)H are important for biomass accumulation and product biosynthesis.

#### Increasing energy capture and delivery

The absorption of solar energy and delivery of electron are limited during the light reactions. Increasing the amount of energy flux by engineering LHCs is performed to enhance the efficiency of light reactions [98, 99]. The state of antenna pigments is important in light reaction as the pigments absorb and transfer the protons to PSII and PSI. During microalgae cultivation, the photoinhibition is amplified as the cell density increases. The antenna pigments function to trap photons and also to block the photons to the deep microalgae in culturing system. As the function to trap photons, the amount and variety of the pigments are the key factors to increase the efficiency of light harvesting. Although high light potentially generates energy sink and strengthens energy dissipation, the increased pigment compositions can alleviate the light and produce high-value products for commercial production [64]. As to avoid photoinhibition, reducing antenna size by genetic modification approaches is promising to improve photosynthetic efficiency in high cell density. The approaches have shown to improve biomass at 11.5–111.5% increase in mass cultures of microalgae [126].

#### Paving lower ATP-cost pathways or providing extra ATP for biomass accumulation

**Fig. 5:**
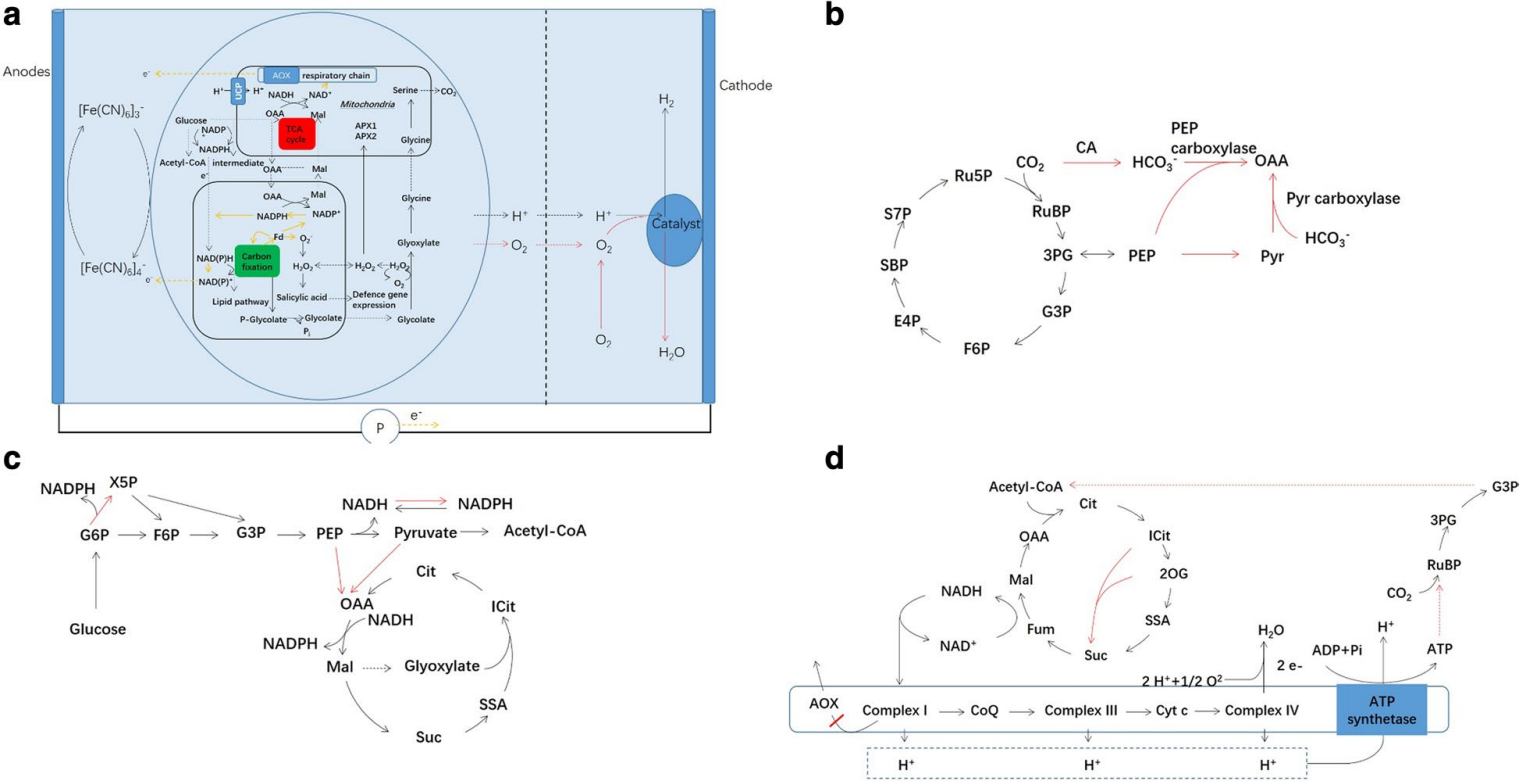
Energy exchange between chloroplast and mitochondria (**a**) and energy rerouting strategies in CO_2_ fixation (**b**), NADPH metabolism (**c**) and TCA cycle (**d**). The red line represents the potential strategies to engineer carbon and energy fluxes, and yellow line represents the electron transport. *RuBP* ribulose 1,5-bisphosphate, *3PG* 3-phosphoglycerate, *F6P* fructose-6-phosphate, *E4P* erythrose-4-phosphate, *SBP* sedoheptulose-1,7-bisphosphate, *S7P* sedoheptulose-7-phosphate, *Ru5P* ribulose-5-phosphate, *X5P* xylulose-5-phosphate, *CA* carboxylases, *PEP* phosphoenolpyruvate, *SSA* succinyl semialdehyde

During CO_2_ fixation of microalgae, the pathway kinetics and ATP requirements are the key features to determine the efficiency of CO_2_ fixation pathways. The poor performance of Rubisco results in slow pathway kinetic and high ATP consumption. As alternative to Rubisco, some attractive carboxylases can be embedded in synthetic CO_2_ fixation pathways with lower ATP costs than the CBB cycle. Phosphoenolpyruvate carboxylase (PEPc) has greater catalytic capacity than Rubisco to fix carbon, with catalytic rate of 3.4–8.4 μmol min^−1^ mg^−1^ to PEPc and 0.9–1.5 μmol min^−1^ mg^−1^ to Rubisco, respectively (Fig. 5b). Several pathways containing these carboxylases seem appealing alternatives to introduce into microalgae, like malonyl-CoA–oxaloacetate–glyoxylate (MOG) pathways based on C-4 plants. The MOG pathways, which do not include any oxygen-sensitive enzymes, have predicted lower ATP costs in aerobic settings. However, the MOG pathways in diatoms are still controversial.

In chloroplast, the products of LEF, CEF and water to water pathway are separated in PS I. Upregulating CEF or water to water pathway in chloroplast is anticipated as the effective strategy to maintain the chloroplast redox state, while the results are undesirable. It has been proved that providing extra ATP by regulating CEF as the main electron transport chain inhibits the process of carbon fixation [127]. Ku et al. [128] engineered an ATP-hydrolysis based driving force module into *Synechococcus* elongatus PCC 7942 to produce 3-hydroxybutyrate at the expense of cell growth. Selecting light to highlight CEF also shows weak enhanced effect in overall process of microalgae cultivation [129]. As a consequence, the plasticity of metabolic pathways to control the intracellular environment is critical to maintain the cell growth. Non-phosphorylation AOX has the capacity to dissipate NADH as heat, and it is also linked to water to water pathway and malate/oxaloacetate shuttle. Therefore, the absence of AOX is promising to rout ATP from mitochondria to chloroplast [8], while the extra ATP might be prone to use in anabolism. The ATP channel between chloroplast and mitochondria is anticipated to be accelerated or enlarged.

Another promising but challenging strategy would be to increase intracellular energy by supplying additional carbon sources. For the advantage of increasing growth rate and promoting product biosynthesis, organic carbon sources like glucose are used to provide more energy for higher rates of growth and respiration, which also change cellular physiology and morphology by affecting metabolic pathways of carbon assimilation and allocation. It has been reported that PP pathway accounted for 90% glucose metabolic flux distribution of *C. pyrenoidosa* in dark using glucose as the sole carbon source, which provided more ATP molecules from glucose than from light in autotrophic an mixotrophic cultivation [130].

#### Forming NAD(P)H sink for lipid biosynthesis

The lipid biosynthesis is related to the substrate concentration (*C*_s_), non-lipid biomass (*C*_*x*_), non-lipid product concentration (*C*_*p*_) and lipid concentration (*C*_*L*_) as follows:

7 $$\frac{{{\text{d}}C_{s} }}{{{\text{d}}t}} = \frac{1}{{Y_{\text{xs}} }}\frac{{{\text{d}}C_{x} }}{{{\text{d}}t}} + \frac{1}{{Y_{\text{ps}} }}\frac{{{\text{d}}C_{p} }}{{{\text{d}}t}} + \frac{1}{{Y_{\text{Ls}} }}\frac{{{\text{d}}C_{L} }}{{{\text{d}}t}} + \mu_{\text{sx}} C_{x}$$

where *Y*_xs_, *Y*_Ls_ and *Y*_ps_ are growth yield on nutrients and production (g/g), *μ*_sx_ is the specific consumption rate of nutrients that maintains cell. Neglecting non-lipid product concentration (*C*_*p*_) and specific consumption rate of nutrients (*μ*_sx_), which show less important in lipid biosynthesis, Eq. (7) is converted as following:

8 $$\frac{{{\text{d}}Cs}}{{{\text{d}}t}} = \frac{1}{{Y_{\text{xs}} }}\frac{{{\text{d}}C_{x} }}{{{\text{d}}t}} + \frac{1}{{Y_{\text{Ls}} }}\frac{{{\text{d}}C_{L} }}{{{\text{d}}t}}$$

9 $$C_{s} = \frac{{C_{x} }}{{Y_{\text{xs}} }} + \frac{{C_{L} }}{{Y_{\text{Ls}} }}$$

10 $$Y = \frac{{C_{L} }}{{C_{s} }} = \frac{{C\frac{{Y_{\text{xs}} Y_{\text{Ls}} }}{{Y_{\text{xs}} - Y_{\text{Ls}} }}}}{{C + \frac{{Y_{\text{Ls}} }}{{Y_{\text{xs}} - Y_{\text{LS}} }}}},$$

where *Y* is the overall process lipid yield and *C *=* L*/(*B *+* L*) is the lipid content. In situation of low lipid content, the *C* is important for commercialization of the lipid production, while in high lipid content for most oleaginous microalgae, *Y* is more significant [131]. Since *Y* is harmonized by the NADPH amounts, forming NAD(P)H sink has the potential to increase the lipid yield and lipid content.

Lipid biosynthesis in particular demands a great deal of NADPH. Forming NAD(P)H sink now is proved to enable commercialization of microbial carbohydrate-based lipid production [131]. In microalgae, NADPH is largely produced from photosynthesis and glycolysis pathways (Fig. 5). Therefore, interference of chloroplastic redox state to increase NADPH may enhance the lipid biosynthesis for a short-term production. For example, restraining water to water pathway by regulating PGR5/PGRL1 proteins makes possible that NADPH sink is formed and high level ROS is increased in chloroplast to promote lipid biosynthesis [132]. Second, glycolysis pathways are desired pathways to regulate the reducing equivalents (Fig. 5c). The overexpression of glucose-6-phosphate dehydrogenase from the PP pathway was reported to have great potential to increase lipid content in microalgae [133, 134]. In addition, activation of PEP/malate/citrate cycle through glyoxylate shunt can convert 1 mol NADH to 1 mol NADPH at a cost of 1 mol ATP. The third strategy is to convert excess NADH directly to NADPH by introducing NADP^+^-dependent glyceraldhyde-3-phosphate dehydrogenase (GPD). However, little information of NADP^+^-dependent GPD is available in microalgae. To regulate the NADPH metabolism, the unbalanced conditions of reducing equivalents may be harmful to cells even induce cellular apoptosis by generating high level of ROS. Therefore, electrochemical system is a novel process to control metabolic pathway under unbalanced conditions of reducing equivalents [135]. Until now, the electrochemical systems in microalgae are used to generate electric current, produce H_2_ and increase product yields, defined as photosynthetic microbial fuel cell, photosynthetic microbial electrolysis cell and electro-fermentation (Fig. 5a). Among these systems, electro-fermentation has been applied in enhancing the final product through the changes in NADH/NAD^+^ [136–138]. Therefore, adding an exogenous cathode in media to accelerate driving forces may be a promising technology to increase products from microalgae.

### New strategies of metabolic engineering in microalgae by coupling carbon and energy fluxes

As described above, advances in algal research have focused on utilizing the topological property of carbon and energy fluxes, which means researches are prone to engineer amphibolic pathways or downstream pathways to influence the targeted pathway. Xiong et al. [7] increased the carbon fixation in CBB cycle by regulating TCA cycle and thus direct CO_2_ to ethylene, Bailleul et al. [8] had successfully “borrowed” ATP from mitochondrial respiratory chain to chloroplastic CBB cycle. However, little information of consolidation contacts between carbon and energy fluxes is available in algal research. The unclear interactions of carbon and energy metabolisms and absence of high-resolution tools for energy changes are the two major limiting factors. Therefore, based on the metabolism and biotechnology reviewed above, we propose several orientations for further microalgae research by consolidating the carbon and energy metabolism:

i. Eliminating energy dissipation and regulating carbon flux in carbon fixation. A mass of researches had proved that energy dissipation was essential for growth of microalgae and plants. Several strategies have proposed to improve carbon fixation and growth by decreasing energy dissipation. It has been proved cutting energy dissipation individually inhibited carbon fixation and cell growth [45]. However, simultaneously applying power supply and knocking out all three respiratory terminal oxidases increased hydrogen production through a bio-photoelectrolysis cell system. Therefore, to recycle the energy dissipated, the drive force to reroute the energy or extra substrate reactions to use the energy are anticipated to maintain the energy balance and redox state (Fig. 5d). The low efficiency of carbon conversion in TCA cycle was proved to limit the efficiency in CBB cycle [7, 101]. Therefore, accelerating the carbon conversion of TCA cycle by eliminati the energy dissipation has potential to increase the rate of carbon fixation.
ii. Accelerating energy flux and regulating carbon flux in product biosynthesis. NADPH and acetyl-CoA, lipid biosynthetic precursors, are important factors to increase substrate-to-product yields. Increasing amounts of the factors by converting glycolytic NADH has proved to be effectively to improve the yield [131]. In addition, to maximize the capture and delivery of electrons from substrate catabolism, the conversion efficiency of NADP^+^ and NADPH in microalgae is required to be accelerated by extra force, which may have less disturbance on redox state than strategy to increase the NADPH amount. Therefore, accelerating energy flux by adscititious electrode has the potential to further increase the yield (Fig. 5a). Simultaneously, regulating carbon flux is anticipated to increase amount and use of NADPH. The promotion of the ratio of NADPH/ATP can be moderated by regulating PP pathway and a metabolic sink of the free fatty acid can be formed by upregulating thioesterase.

As more information of the fluxes is obtained, various technologies such as Electrochromic Shift (ECS) and fluorescent probes are gushed to boost CO_2_ fixation and yields of biofuels and chemicals from microalgae. The non-invasive and continuous properties of an index in live microalgal cell are credible to reveal the nature of cellular metabolism. ECS signals have been proposed for a long time to reflect conditions of energy and pigment in algal cells [139]. ECS represents a shift in the absorption spectra of some photosynthetic pigments that are induced by electric field in light-dependent reaction. Normally, ECS is expressed as follows:

11 $${\text{ECS}} = (\mathop \to \limits_{F} ,\mathop \to \limits_{{F^{2} }} ),$$

where $\mathop \to \limits_{F}$ is electric field. That means ECS is composed by ECS_lin_ and ECS_quad_ [140], the linear relation between ECS and energy, or pigment is impractical in overall process of algal growth. In addition, recent developments of encoded fluorescent sensors make possible to observe dynamic metabolism in living cell. The H_2_O_2_ sensor HyPer provides another strategy to assess the interaction between chloroplast and mitochondria in microalgae [141]. Therefore, development of new optical probes has great potential in microalgal metabolism like NADPH sensor [142]. These technologies will help to open the field to more systematic application in microalgae. In general, other subjects such as electrochemistry and analytical chemistry are promising for the high resolution.

## Industrial products from microalgae and maximal biomass value

Although tremendous approaches have successfully promoted the biomass and product yields, commercialization of the functional components still remains a challenge. Recent techno-economic analysis work has suggested cost reductions for biofuel economical viability of microalgae was essential, while exceedingly difficult [126]. The development of high-value products is the traditional path to reduce the cost by increasing the inherent value of microalgal biomass. The strategies to improve cell growth, carotenoids and lipid accumulation were discussed above. Another path is to optimize the cultivation system by reducing the input of energy and high-value substance.

### Valuing the product: market

In the economic analysis, the three storage carbon substances: protein, carbohydrate and lipid are the main products from microalgae that are exploited in various market scenarios. As the extraction of functional proteins is high cost with low revenue in cultivation process, the microalgal cells are usually broken as animal feed [143]. The prices of animal feed are calculated with a broad correlation with the protein content [144]. The protein-rich microalgae, such as *C. pyrenoidosa*, are the promising strains in animal feed that can get the value of 750 $ tonne^−1^ [144]. The carbohydrate from microalgae has got increasing attention nowadays due to its renewable and environmental friendly advantages to produce alcohol and methane. However, to obtain high revenue from the biomass, the low carbohydrate content is profitable as its price is merely around 1100 $ tonne^−1^ [143]. The digestion of carbohydrate, therefore, is usually performed after the extraction of lipid for the recyclable use of cell debris. Since lipid and carotenoid are the profitable products from microalgae, they are used to investigate the potential of the microalgae-derived products in biofuels and food additives markets [126]. The lowest values of both lipid and carotenoid are in biofuels market. Only considering the cost for heat in microalgae cultivation, previous study showed a cost per unit of dry cell weight of 4000 $ tonne^−1^ for the cultivation. However, the revenue from the lipid in biofuels was merely around 200 $ tonne^−1^ [143]. The loss can be offset by the high revenue from food additives market. The prices of lipid and carotenoid in this market are around eightfold and 135 fold higher than that in biofuels, respectively. Orchestration of process-compatible products, therefore, is an alternative to maximize biomass value, as a means to resolve the potential conflict between revenues of high valuable products and biofuels [93].

### Application of strategies for maximal biomass value

The comprehensive understanding of carbon metabolism and energy conversion of microalgae will provide preferable orientations to design the cultivation processes. The enhancement of biomass concentration is always encouraged for the researchers, not only in microalgae production, but also in waste treatment. To some extent, the researchers make great efforts to keep the biomass increasing, and then nutrient loading amount from waste and bioproduct from microalgae are continuously promoted. The lacking understanding of key limiting factors during the cultivation processes, which can be identified by carbon metabolism and energy conversion, is the major difficulty for the researchers to increase the biomass concentration. This might be one of the reasons that researches usually obtain sub-optimum biomass concentration at the industrial scale. For example, to overcome the nutrient limitation during cultivation processes, fed-batch culture is usually performed based on the microalgal characteristics of specific growth rate and nutrient yield on biomass. These characteristics of a specific strain, however, are fluctuant under different cultivation conditions, which are supposed to determine based on the cultivation systems. To increase the efficiency of light conversion, various bioreactors can be assembled to shorten the optical path. The fermenter is a stable and easy system to control the cell growth, while its low transmittance of light largely limits the final biomass concentration. Therefore, the flat plate bioreactor can be assembled to fermenter with the 12 h day at plate bioreactor and 12 h dark at fermenter.

The low biomass concentration in mass cultivation is still the major challenge for the microalgae production. Indeed, the lipid and carotenoid productivities are largely dependent on biomass concentration [34, 93]. Although one-stage cultivation is capable of increasing the product yield with less input of energy and equipment, the economic analysis still reveals multi-stage cultivation is more profitable in microalgae production [34]. Consequently, the approaches to optimize growth condition and satisfy the requirement of nutrient and light are always the first and key steps in most microalgae production. These approaches can result in several-fold or even more increase of the biomass concentration, with similar cost of the traditional cultivation. The strategies to enhance photosynthetic efficiency, availability of light and absorption capability of nutrient are always promising approaches to increase the biomass value by addressing the environmental issues. These strategies are focused on the increased biomass concentration by utilizing the extracellular substance and energy. The approaches such as using fed-batch culture and improving light conditions by mixing lights are promising options to maximize the biomass productivity. Engineering strategies on CBB and TCA cycles to increase the photosynthetic efficiency can be also used to overcome the bottleneck of mass cultivation. Until the biomass concentration no longer increased in the suitable conditions, the carbon partitioning in cell is required to be regulated for the high biomass value. However, to maximize the product content in microalgae, stress-based strategies and metabolic engineering focusing on change of the carbon partitioning may lead to growth retardation or even cell death. As mentioned above, the revenue from protein is lower than that from carbohydrate, lipid and carotenoid. For the interest setting, conversion of protein into these substances, especially lipid and carotenoid, is the promising option to increase the biomass value. With the same cell weight, N-starvation promoted the lipid value in both biofuel and food additive markets, while largely damaging the carotenoid value. The application of excess light in food additive market was profitable, as it promoted the lipid and carotenoid values [93]. However, in the single stressed condition, microalgae always pave the most elevated carbon availability into carbohydrate, not lipid, which largely limits the value. Therefore, more severe environment conditions are usually created to increase the lipid and carotenoid contents. Combination of excess light and other stresses is the promising strategy to increase the value in food additive market, while the use of light should be avoided in biofuel field as it decreases the oil quantity. As an alternative, multi-stage cultivation can be used to accumulate carbohydrate first and then to degrade the carbohydrate to lipid. Regulation of starch biosynthesis and degradation has the potential to achieve more desirable products such as lipid and carotenoid. The supply of carbon molecules can be from intracellular protein degradation and extracellular media. Under stressed conditions, the main source of starch is from protein, while the growth rate is mostly limited and consequently the total production and productivity are decreased. The negative effects mitigate the application of stress-based strategy. Consequently, the application of two-stage cultivation and fed-batch culture will greatly enhance the carbon molecules in starch. To reduce the time cost, it would be interesting to consider ways to accelerate the conversion rate from protein to the products. Sun et al. [34] provided blue light on *H. pluvialis* at the stage of astaxanthin accumulation and shortened the cultivation time with high astaxanthin content. In short, coordination of multi-stage cultivation with engineering and stress-based strategies occupies significant positions in a long term.

Another strategy is to apply microalgae in waste treatment, which can offset the cultivation cost by addressing the environment issues [130]. The main nutrients for the cultivation of microalgae are carbon, nitrogen and phosphorous. Several industrial and life wastes like food waste and aquaculture wastewater contain abundant nutrients, which are essential for microalgae growth. These wastes are shown to be appropriate nutrient source in heterotrophic cultivation of microalgae. The biomass and product yield are acceptable for industrial production. Using wastes for the production of lipid, carbohydrates and proteins, factors of nutrient composition, energy conversation and nutrient concentration directly affect the biomass accumulation and product biosynthesis. For example, lipid-extracted microalgal biomass residues (LMBRs) and molasses are shown to be superior to kelp waste for increasing biomass and lipid of *Chlorella* sp. [145, 146]. In addition, depending on the initial nutrient compose in waste and the processing conditions, extra nutrient like glycerol is anticipated to be supplied to optimize the natural medium and also to accelerate lipid biosynthesis [147]. As an effective technology, pretreatment of wastes is promising to increase the efficiencies of nutrient and energy conversation [148].

## Conclusion

Understanding the storage carbon metabolism and energy conversion of microalgae to produce bioproducts and biofuels can bring to light the key barriers, which are the key hinges to guide prospective biomass component and productivity for the commercial viability. The designs of cultivation modes and methods to maintain energy (primarily from light) and nutrient balances have the objective of creating a suitable environment for cell growth and the designs to disturb the balances aim at forming the stressed environment for product biosynthesis. To achieve more desirable high-value products, coordination of multi-stage cultivation with engineering and stress-based strategies occupies significant positions in the long term.
