# Correction to: High-value biomass from microalgae production platforms: strategies and progress based on carbon metabolism and energy conversion

**DOI:** 10.1186/s13068-018-1253-2

**Published:** 2018-09-19

**Authors:** Han Sun, Weiyang Zhao, Xuemei Mao, Yuelian Li, Tao Wu, Feng Chen

**Affiliations:** 1 Institute for Food & Bioresource Engineering, College of Engineering, Peking University, Beijing, 100871 China; 2 BIC-ESAT, College of Engineering, Peking University, Beijing, 100871 China

## Correction to: Biotechnol Biofuels (2018) 11:227 10.1186/s13068-018-1225-6

Following publication of this article [1] the authors noted that there is a redundant icon in Fig. 1. A replacement Fig. 1 can be found here.

**Fig. 1:**
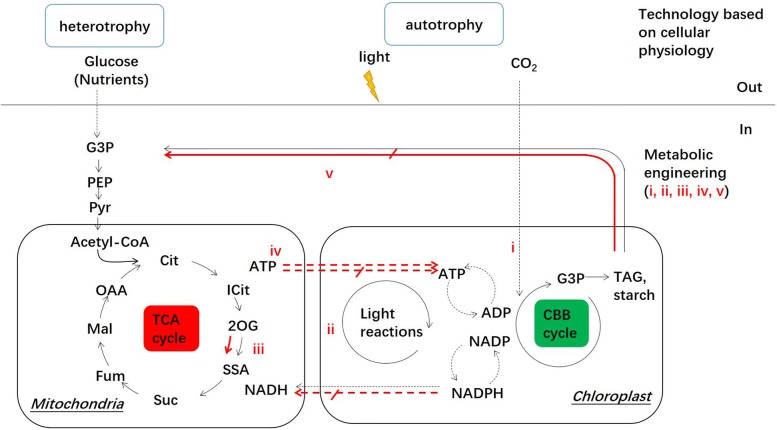
Carbon metabolism and energy conversion in glycolysis, CBB and TCA. In carbon fixation of microalgae, the metabolic engineering is traditionally focused on efficiency of CBB cycle and light reactions in chloroplast (i and ii). Advances have noticed that engineering TCA cycle increases the carbon fixation (iii and iv). Coupling carbon and energy fluxes have proposed as the trends of metabolic engineering in microalgae (v). The traditional technology based on cellular physiology focuses on conditions of CO_2_, light and nutrient in microalgae cultivation. The carbon metabolites: *G3P* glyceraldehyde-3-phosphate, *PYR* pyruvate, *PEP* phosphoenolpyruvate, *OAA* oxaloacetate, *MAL* malate, *FUM* fumarate, *SSA* succinyl semialdehyde, *SUC* succinate, *2OG* 2-oxoglutarate, *CIT* citrate, *G3P* glyceraldehyde 3-phosphate
